# Assessing whether ad hoc clinician-generated patient questionnaires provide psychometrically valid information

**DOI:** 10.1186/s12955-020-01287-2

**Published:** 2020-03-03

**Authors:** Minji K. Lee, Jeffrey R. Basford, Allen W. Heinemann, Andrea Cheville

**Affiliations:** 1 Kern Center for the Science of Health Care Delivery, Mayo Clinic, 200 First Street SW, Rochester, MN 55905 USA; 2 Department of Physical Medicine and Rehabilitation, Mayo Clinic, Rochester, MN USA; 3 Center for Rehabilitation Outcomes Research, Shirley Ryan AbilityLab, and the Department of Physical Medicine and Rehabilitation, Northwestern University, Chicago, IL USA

**Keywords:** Patient reported outcome, PRO, Psychometrics, Past medical history, Social history, Item response theory, Factor analysis, Quality of life, Review of systems

## Abstract

**Background:**

The provision of psychometrically valid patient reported outcomes (PROs) improves patient outcomes and reflects their quality of life. Consequently, ad hoc clinician-generated questionnaires of the past are being replaced by more rigorous instruments. This change, while beneficial, risks the loss/orphaning of decades-long information on difficult to capture/chronically ill populations. The goal of this study was to assess to the quality of data retrieved from these legacy questionnaires.

**Methods:**

Participants included 8563 patients who generated a total of 12,626 hospital admissions over the 2004–2014 study period. Items used to screen for issues related to function, mood, symptoms, and social support among patients with chronic disease were identified in our medical center’s patient information questionnaire. Cluster and exploratory factor analyses (EFA) followed by multidimensional item response theory (MIRT) analyses were used to select items that defined factors. Scores were derived with summation and MIRT approaches; inter-factor relationships and relationships of factor scores to assigned diagnostic codes were assessed. Rasch analyses assessed the constructs’ measurement properties.

**Results:**

Literature review and clinician interviews yielded four hypothesized constructs: psychological distress/wellbeing, symptom burden, social support, and physical function. Rasch analyses showed that, while all had good measurement properties, only one, function, separated individuals well. In exploratory factor analyses (EFA), 11 factors representing depression, respiratory symptoms, musculoskeletal pain, family support, mobility, activities of daily living, alcohol consumption, weight loss, fatigue, neurological disorders, and fear at home were identified. Based on the agreement between EFA and cluster analyses as well as Cronbach’s alpha, six domains were retained for analyses. Correlations were strong between activities of daily living and mobility (.84), and moderate between pain and mobility (.37) and psychological distress (.59) Known-group validity was supported from the relationships between factor scores and the relevant diagnostic code assignments (.12 to .20).

**Conclusions and discussion:**

Items from ad hoc clinician-generated patient information questionnaires can be aggregated into valid factors that assess supportive care domains among chronically ill patients. However, the binary response options offered by many screening items limit their information content and consequently, as highlighted by Rasch analyses, their ability to meaningfully discriminate trait levels in these populations.

## Introduction

Patient reported outcomes (PROs) have gained acceptance as an important component of clinical care. This acceptance is likely to grow as an increasing body of research suggests that the provision of PRO data to clinicians at the point of care can improve outcomes and even survival [1, 2]. Such findings support the idea that these tools can accurately reflect constructs such as symptom burden and psychological well-being, that can have a broad effect on a patient’s functional status, longevity, and quality of life. In addition, emerging associations between PROs and unplanned health care utilization, e.g., 30-day readmissions, suggest that they may also have a role in improving medical care, reducing health care inefficiencies, and the delivery of supportive care [3].

The collection of PRO data is not new. Many healthcare institutions have collected PROs for decades with the use of empirically developed, facility-specific questionnaires. While ad hoc instruments are used frequently for clinical screening and to fulfill review of system requirements, their use is increasingly being orphaned by the widespread recognition of the need for psychometric rigor in instrument development [4, 5]. Such standards have resulted in new measures and improved PRO data quality. However, as a consequence of this advance, we now have vast sets of ad hoc PRO-based data that at best may not be used to their full advantage and, at worse, be discarded.

Although these legacy data sets lack the psychometric vetting now expected of PROs, they have many strengths. Specifically, they are often huge, have high rates of completion due to institutional mandates, span decades, and include longitudinal information on difficult to capture populations. In particular, many have a high proportion of chronically ill patients who are generally under-represented in medical studies, and may, therefore, be of value in informing the delivery of geriatric, rehabilitative and supportive care services.

In the case of our institution, the systematic collection of institution-wide PRO information began in the early-1990s in the form of patient responses to a “Current Visit Information” (CVI) questionnaire. The instrument is completed at six-month intervals, and queries patients about their function, symptoms, habits, and psychological well-being, as well as other issues such as medication use and allergies. While the items have never been formally validated, the questionnaire, with a few minor variations, has served as a point-of-care clinical resource for over 20 years. The result has been the accumulation of a vast amount of information: the past decade alone includes more than 4 million assessments from over a million patients.

Our long-term goal is to establish whether patient-reported data systematically collected in outpatient settings with a non-validated instrument can be used to improve and individualize the services delivered to hospitalized patients, improve long-term outcomes, reduce post-acute care requirements, and prevent 30-day readmissions. The goals of this initial paper were more modest and two-fold. The first was to describe the methods that we used to identify, cluster, and score items related to patients’ care needs that they reported before hospitalization. The second was to outline the utility and drawbacks of these methods as a guide to others who might be planning to assess the value of their institution’s “homegrown” questionnaires.

## Methods

The study was approved by the Mayo Clinic Institutional Review Board. Informed consent was waived as it utilized de-identified data collected from patients as part of routine clinical care.

### Sample

The sample was derived from patients who were admitted to one of the Rochester-based Mayo Clinic hospitals for coronary artery disease (CAD), chronic obstructive pulmonary disease (COPD), myocardial infarction (MI), congestive heart failure (CHF), and/or pneumonia over a 10-year period between May 2004 and May 2014. Exclusion criteria were residence more than 130 km (80 miles) away (to minimize loss of data due to hospitalization elsewhere), lack of a research authorization, death or an age 18 years at any time during the study period. We selected records for patients who provided a questionnaire within 6 months prior to a hospital admission and completed at least 70% of the 84 items.

### Identification of unusable CVI items

As with many forms developed for clinical use, our institutions’ questionnaire evolved over time with respect to item inclusion, wording, and response options. Therefore, we reviewed all versions in order to eliminate items that had been altered substantially. Four forms were used over the study period. Form A, completed by 1002 patients, was used until early 2005. Form B (the dominant and current questionnaire) evolved from Form A, and was completed by 7988 patients. The Spanish language Form C, which was identical in content to Form B, was completed by 9 patients. An online form, D, was used from 2009 to 2014 by 41 patients, with content similar to Form B.

Forms B and C consist of 4 sections that include items related to: 1) Review of Symptoms (fatigue, fever, weight gain/loss, appetite, depressed mood, etc.); 2) Social History (living situation, ethanol and tobacco use); and 3) Functional status (activities of daily living, need for and availability of assistance with home care, mobility, and use of assistive devices). All items potentially related to underlying latent traits were included in subsequent steps to identify relevant item clusters. All, excepting a single item, “*Can you climb two flights of stairs without stopping to rest?*.” offered binary response options.

### Identification of constructs related to care needs, disposition, and re-admission risk

Several methods were used to identify item clusters that might represent constructs relevant to potential care needs. First, we conducted a literature review on the predictors of 30-day hospital readmission and the need for institutionalized post-acute care. We found that, among the many predictors, those that could be assessed using CVI data included comorbidities, symptoms, functional impairments, and demographic/social variables (e.g., living arrangements, and social support) [6–11].

Second, clinician content matter experts; hospitalists, palliative care, and rehabilitation medicine physicians with > 10 years of practice experience, initially identified 81 items in the CVI (Supplement 1) that assessed aspects of potentially actionable constructs that had been identified through the literature review. We hypothesized that four constructs associated with hospital care needs (psychological distress/wellbeing, symptom burden, social support, and physical function0 could be estimated by aggregating and scoring the CVI items. Following hypothesis generation, the item pool was reduced from 81 to 56 items because 25 items had less than 10% response rates, while 56 retained items had response rates > 70 + %.

### Addressing missingness

Missing values were filled in with a nonparametric, mixed-type imputation method using Random Forest algorithm which has been validated in similar studies [12, 13].

### Determination of whether hypothesized constructs were supported by CVI items

Once the four constructs (psychological distress/wellbeing, symptom burden, social support, and physical function) were identified, we used a two-pronged approach to evaluate whether the CVI items could be aggregated. First, we used Rasch analyses to assess whether the four hypothesized constructs have good measurement properties. Then, we investigated whether different groups of items could be obtained using statistical methods such as exploratory factor analysis and cluster analysis. For the latter approach, we evaluated the fit of items to the new constructs using confirmatory analyses such as multidimensional IRT.

#### Part 1. Rasch analyses

Rasch analysis rests on the assumption/requirement that a set of items measures one underlying construct and that the items form a hierarchy from easiest to hardest to endorse. In our case, we hypothesized that the 56 items comprise four constructs; psychological distress, symptom burden, social support, and physical function. Using the Rasch model, we determined whether the CVI items and respondents were separated adequately along a logit scale for each hypothesized construct. We also examined separation reliability for items and respondents to provide a measure of the degree to which the respondents or items are separated. To address both item fit and person fit (consistency), we estimated fit statistics such as the outfit mean statistics, the unweighted mean square residual differences between observed values and expected values [14]. WINSTEPS 4.0.1 [15] was used for these analyses.

#### Part 2. Exploratory factor analysis/cluster analysis followed by confirmatory factor analysis and multidimensional item response theory analysis

##### Exploratory factor analysis

Rather than assuming particular item-to-construct relationships, we created constructs using statistical methods. We created two equal-sized random samples, one for exploratory analyses, and the other for confirmatory analyses of the constructs.

We defined the constructs using exploratory factor analysis (EFA) [16]. The number of factors extracted was determined by parallel analysis [17]. Maximum likelihood nonlinear EFA with promax rotation was performed to approximate the independent-clusters structure. The strengths (correlations) of item-to-factor loadings were evaluated to identify meaningful clusters using the cutoff of |.32| [18], which represents 10% of the shared variance between the item and factor. Coefficients greater than |0.60| were considered large, and those of |0.35|-|0.59| moderate [19].

##### Cluster analysis

We assessed the robustness of the factor structure identified with EFA using cluster analysis to produce an operational classification. We performed a hierarchical agglomerative cluster analysis to partition the sample into homogeneous classes using Ward’s method applying squared Euclidean Distance as the distance measure. A hierarchical tree diagram (i.e., a dendrogram) was produced to show the linkage points.

### Confirmation and refinement of constructs

Using the second half of the sample, we applied two approaches to obtain fit statistics for the factor structure identified through the EFA and cluster analyses.

#### Multidimensional 2-parameter item response theory (MIRT) analysis

We performed MIRT analysis for several reasons. First, it permitted an assessment of item position along the unidimensional trait continua which allowed us to evaluate whether items discriminated in the trait range relevant to hospitalized patients. Second, several constructs were supported by only a limited number of items, and MIRT approaches enable “borrowing” of information from correlated constructs.

MIRT confirmatory nonlinear factor analysis was performed using the normal Ogive Harmonic Analysis robust method as described by McDonald [20]. Fit indices such as root mean square residual (RMSR), root mean square error of approximation (RMSEA), and the Tanaka goodness-of-fit index were used to evaluate the model fit [21, 22]. We obtained the item parameters (i.e., item discrimination and item difficulty) from the IRT analyses noted above. The item difficulty parameter typically ranges from − 3 to 3, and decreases in value as the item becomes easier to endorse.

#### Final constructs and items

The investigative team and clinician content experts reviewed the results of the confirmatory factor, MIRT, and Rasch analyses. Final constructs and their constituent items were determined through a Delphi consensus process.

### Scoring

Several scoring approaches were used and compared. First, constructs were scored using the item parameters from the MIRT model using a Metropolis-Hastings Robbins-Monro estimation [23–25]. Second, summed scores were calculated from the raw data. Third, summed item scores weighted by factor loadings from confirmatory factor analyses were developed. Correlations between the results of the different scoring strategies were estimated. Analyses were performed using R version 3.3.0, R package “sirt” for IRT estimation [26], and flexMIRT® for multidimensional IRT scoring [27].

### Discriminant and convergent validit and known group comparisons

We tested the validity of the summed construct scores over an interval spanning 1 year before and after hospital admission. Specifically, we estimated correlations between the different construct scores, as well as comparisons of the scores associated with the assignment of ICD-9 codes for diagnoses associated with the hypothesized constructs: anxiety (300), pulmonary symptoms (786, 460–519, 786), musculoskeletal pain (710–739, 338.2), and arthritis (714–716). For known-group validity, we used Mann-Whitney U tests to compare the factor scores from the groups with or without the diagnoses.

## Results

### Sample

The sample consisted of 8563 patients who generated a total of 12,626 hospital admissions over the period of the study (Table 1**).** In brief, the sample included more men (62%) than women with a median age of 73 years (interquartile range (IQR 62 to 82)). Four diagnoses (CAD 31%, CHF 21%, pneumonia 21%, and MI 19%) accounted for more than 90% of the admissions, while 8% involved COPD. About 70% of the admissions were charted as “emergency”, “urgent” or “semi-urgent” and 30% as “non-emergent.” The mean distance between patients’ residence and hospital was 40 km (25 miles). About three-fourths (75%) of the admissions were discharged home, 5% home with home health care, and 20% post-acute care. Roughly 64% had some high school or general equivalency diploma (GED) as their highest level of education; 37% had gone to at least some college or more. Nearly two-thirds (65%) were married, 18% were widowed, most were retired (57%), and 20% were employed.

**Table 1:** Demographic and clinical characteristics of the study participants

*N*	8,563
Age, min, Q1, median, Q2 max	19, 62, 73, 82, 104
Sex (female), *n* (%)	3,265 (38%)
Race/ Ethnicity, *n* (%)	
White	8,002 (93%)
Black/ African American	86 (1%)
Asian	68 (0.8%)
American Indian/ Alaskan Natives	17 (0.2%)
Hispanic	30 (0.4%)
Marital status, *n* (%)	
Married	5,535 (65%)
Single/never married	728 (9%)
Divorced/ separated	714 (8%)
Widowed	1,558 (18%)
Highest level of school completed	
8th grade or less	881 (10%)
Some high school, but did not graduate	672 (8%)
High school graduate or GED	3,262 (38%)
Some college or 2-year degree	1,747 (20%)
4-year college graduate	686 (8%)
post graduate studies	736 (9%)
Employment status	
Employed	1,397 (16%)
Self-employed	354 (4%)
Retired	4,907 (57%)
Unemployed	264 (3%)
Work disabled	383 (4%)
Full-time homemaker	200 (2%)
Disease category, *n* (%)	
CAD	2,662 (31%)
CHF	1,791 (21%)
COPD	652 (8%)
MI	1,651 (19%)
Pneumonia	1,807 (21%)
Admit type	
Trauma Center	3 (0.03%)
Emergency	8,007 (59%)
Urgent	849 (10%)
Semi-urgent	47 (0.5%)
Routine (Reserved)	2,576 (30%)
Distance in miles, mean (SD)	25.3 (19.6)
Discharge disposition, *n* (%)	
Home	6,413 (75%)
Home with health care	470 (5%)
Facility	1,680 (20%)

*Note.* Emergency – The patient required immediate medical intervention as a result of severe, life threatening, or potentially disabling conditions. Generally, the patient was admitted through the emergency room. Urgent – The patient required immediate attention for the care and treatment of a physical or mental disorder. Generally, the patient was admitted to the first available and suitable accommodation. Trauma Center – visits to a trauma center/hospital as licensed or designated by the State or local government authority authorized to do so, or as verified by the American college of Surgeons and involving a trauma activation

### CVI data characteristics

The 56 items used in the analyses ranged in data completeness from 73 to 95% with a mean of 87%. On average, the questionnaires had been administered 57 (median 37) days prior to hospital admission. The degree of missingness did not differ significantly across disease- or demographically-defined subgroups. The mean interval between a questionnaire’s completion and hospitalization was about 1 month longer for the CHF, COPD and pneumonia groups (2.5 months) compared to those with CAD or MI (1.5 months). This interval was also slightly longer for emergency and urgent admissions (2 months) compared to those rated as non-emergent care admissions (1.5 months).

Table 2 lists summary statistics of the responses to the 56 items. All items were binary with 1 indicating “yes”, except one, which measured function, “Can climb two flights of stairs without stopping to rest?” A response of 0 on this item indicated no difficulty, 1 some difficulty, and 2 inability.

**Table 2:** Descriptive statistics of item responses

Content of items and the name of the constructs (bolded) that the items were hypothesized to measure	Mean (SD)	Missing responses (%)
Psychological Distress/ Well being		
1. Do relatives/ friends worry about your alcohol consumption?	0.03 (0.2)	493 (6%)
2. Recurring thoughts of death or suicide?	0.01 (0.1)	616 (7%)
3. Have you ever felt the need to cut down on your alcohol consumption?	0.06 (0.2)	438 (5%)
4. Have you felt anxious or nervous?	0.10 (0.3)	615 (7%)
5. Have you felt restless and irritable?	0.06 (0.2)	615 (7%)
6. Have you felt sad most of the time?	0.04 (0.2)	616 (7%)
7. Have you had difficulty concentrating?	0.06 (0.2)	616 (7%)
8. Have you had little interest or pleasure in relationships or activities?	0.06 (0.2)	616 (7%)
Symptom Burden		
9. Bothered with coughing?	0.22 (0.4)	20 (0.2%)
10. Bothered with shortness of breath?	0.40 (0.5)	20 (0.2%)
11. Bothered with wheezing?	0.13 (0.3)	20 (0.2%)
12. Difficulty with pain?	0.36 (0.5)	1,893 (22%)
13. Had abnormal swelling in the legs or feet?	0.22 (0.4)	28 (0.3%)
14. Back pain	0.22 (0.4)	28 (0.3%)
15. Changes in bowel movement	0.03 (0.2)	14 (0.2%)
16. Difficulty moving an arm or leg	0.16 (0.4)	29 (0.3%)
17. Difficulty swallowing	0.07 (0.3)	14 (0.2%)
18. Difficulty with leaking urine	0.11 (0.3)	13 (0.2%)
19. Diminished hearing	0.14 (0.3)	28 (0.3%)
20. Excessive daytime drowsiness	0.13 (0.3)	616 (7%)
21. Fatigue	0.25 (0.4)	615 (7%)
22. Joint pain	0.25 (0.4)	28 (0.3%)
23. Joint swelling	0.07 (0.3)	29 (0.3%)
24. Loss of appetite	0.09 (0.3)	615 (7%)
25. Muscle pain	0.15 (0.4)	29 (0.3%)
26. Nausea	0.08 (0.3)	13 (0.2%)
27. No symptom(s)	0.87 (0.3)	616 (7%)
28. Problems falling asleep	0.18 (0.4)	615 (7%)
29. Significant Headaches	0.10 (0.3)	28 (0.3%)
30. Significant problems with constipation	0.10 (0.3)	13 (0.2%)
31. Significant problems with diarrhea	0.07 (0.3)	13 (0.2%)
32. Weight gain of more than 10 pounds	0.05 (0.2)	39 (0.5%)
33. Weight loss of more than 10 pounds	0.07 (0.2)	38 (0.4%)
Social Support		
34. Ever fearful for your own safety?	0.02 (0.1)	1,255 (15%)
35. Ever feel afraid in your own home?	0.02 (0.1)	1,123 (13%)
36. A living will or other advance directive?	0.45 (0.5)	1,706 (20%)
37. Family/friends who can provide assistance with homecare needs	0.75 (0.4)	554 (6%)
38. Divorced or widowed in the past year?	0.04 (0.2)	726 (8%)
39. Assisted Living-created from “Current living arrangements”	0.11 (0.3)	322 (4%)
40. Committed relationship-created from “Current relationship status”	0.64 (0.5)	343 (4%)
41. Living with family-created from “With whom do you live?”	0.70 (0.5)	456 (5%)
Function		
42. Can climb two flights of stairs without stopping to rest?	1.05 (0.8)	1,465 (17%)
43. Depend on any assistive devices (wheelchair, cane) or assistance from other people to perform activities important in your daily life?	0.34 (0.5)	1,032 (12%)
44. Difficulty bathing by yourself	0.15 (0.4)	739 (9%)
45. Difficulty climbing stairs by yourself	0.38 (0.5)	738 (9%)
46. Difficulty dressing by yourself	0.12 (0.3)	739 (9%)
47. Difficulty eating by yourself	0.03 (0.2)	739 (9%)
48. Difficulty housekeeping by yourself	0.23 (0.4)	738 (9%)
49. Difficulty performing these activities by yourself—None	0.48 (0.5)	739 (9%)
50. Difficulty taking medications by yourself	0.15 (0.4)	739 (9%)
51. Difficulty transportation by yourself	0.14 (0.3)	739 (9%)
52. Difficulty using the toilet by yourself	0.07 (0.2)	739 (9%)
53. Difficulty walking by yourself	0.26 (0.4)	738 (9%)
54. Difficulty getting in and out of bed by yourself	0.09 (0.3)	1,354 (16%)
55. Difficulty preparing meals by yourself	0.17 (0.4)	1,354 (16%)
56. Tendency to fall easily	0.08 (0.3)	616 (7%)

### Part 1. Rasch analyses evaluation of the measurement properties of the hypothesized constructs

Unidimensional Rasch models were fit for the four hypothesized constructs, each having at least 8 items. The infit mean square for persons of 0.88 to 1.00 suggested that, overall, the four scores allow for valid measurement of each person. The item separation reliabilities (Table 3) ranged from 0.99 to 1 depending on constructs, which indicated that each of the four measures can distinguish a wide range of its respective construct.

**Table 3:** Item measurement results

	Psychological Distress	Symptom Burden	Social Support	Function
Measures				
Mean	0.00	0.00	0.00	0.00
SD	0.89	1.61	2.46	2.12
N	8	25	8	15
OUTFIT				
Mean	0.97	1.03	1.30	1.53
SD	0.25	0.49	0.40	2.28
> 2 (count)	0	1	1	1
INFIT				
Mean	1.00	1.00	0.99	0.97
SD	0.19	0.06	0.10	0.33
> 2 (count)	0	0	0	0
PBIS^a^ (Counts)				
<.00	0	0	0	0
0-0.2	0	1	4	0
Reliability of Separation	0.99	1.00	1.00	1.00

^a^Point-biserial correlation is a measure of item discrimination

Additionally, the overall outfit mean squares of 0.97 to 1.30 for psychological distress, symptom burden, and social support suggested that the items in the scale are working well together to define their construct. The outfit mean square for function was 1.53, which does not degrade the measurement system but is unproductive for construction. Upon close examination of the outfit statistic for each item, the “tendency to fall” item had an outfit statistic of 9.90, and thus should be removed from function domain.

**Fig. 1:**
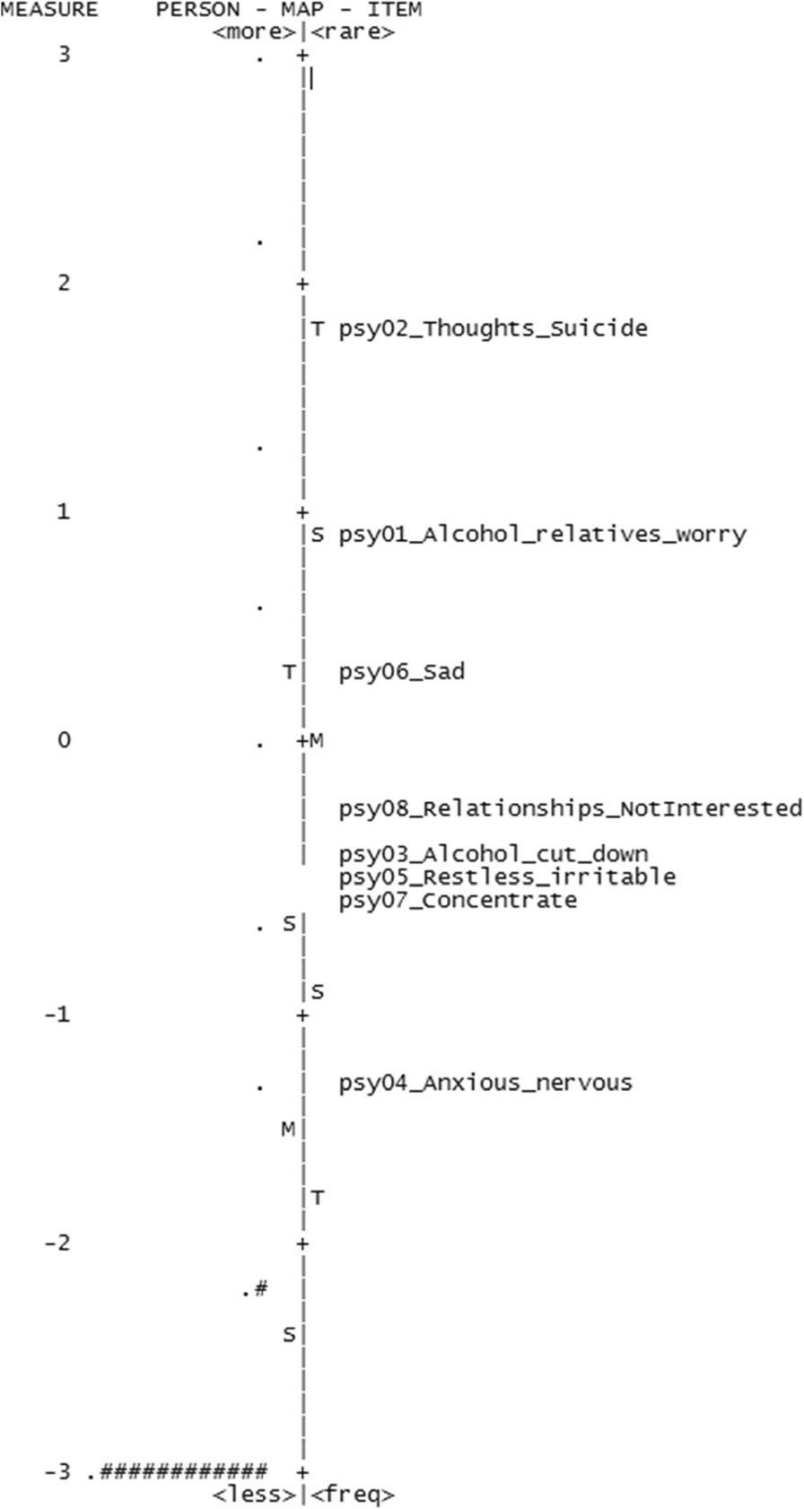
Variable map for psychological distress

**Fig. 2:**
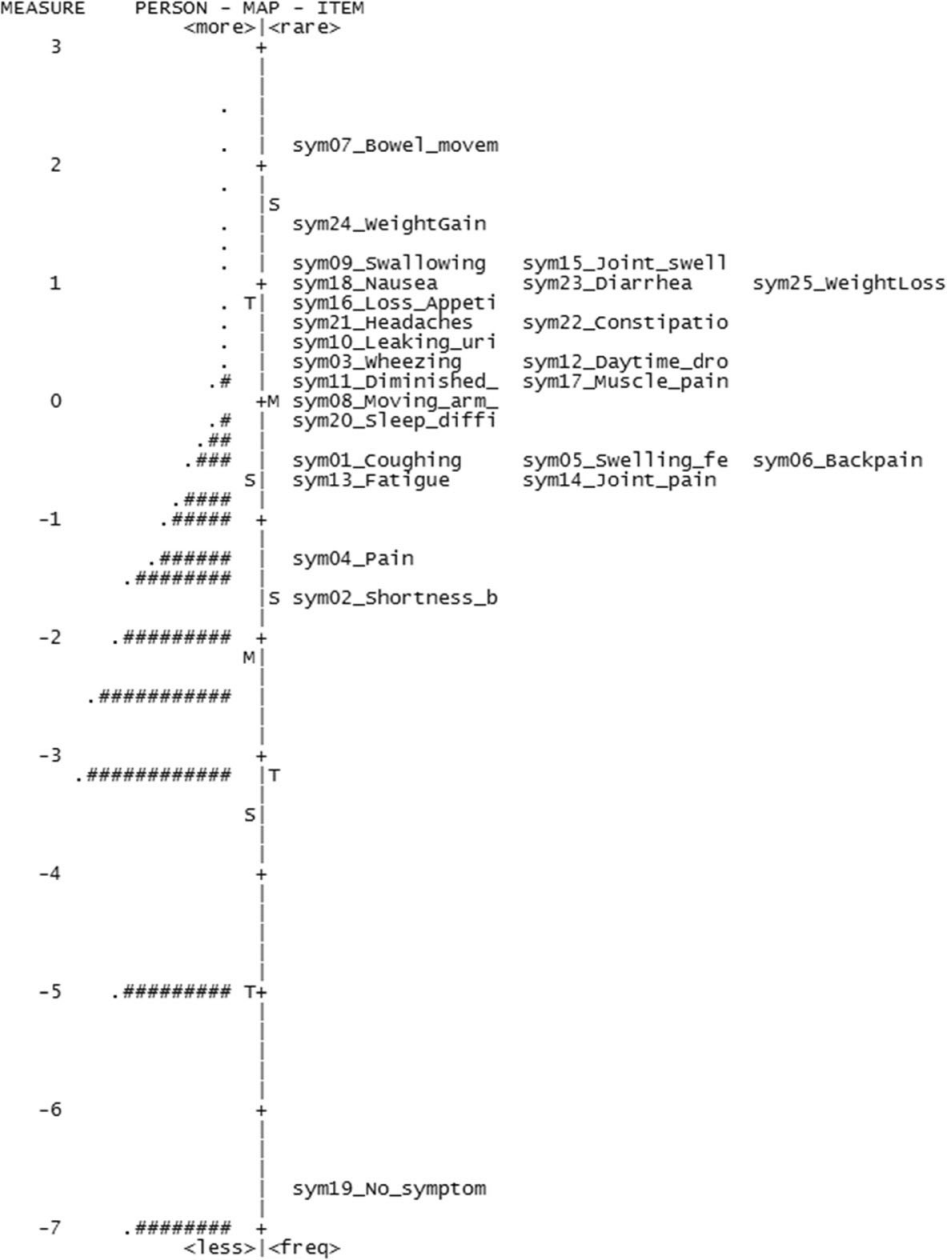
Variable map for symptom burden

**Fig. 3:**
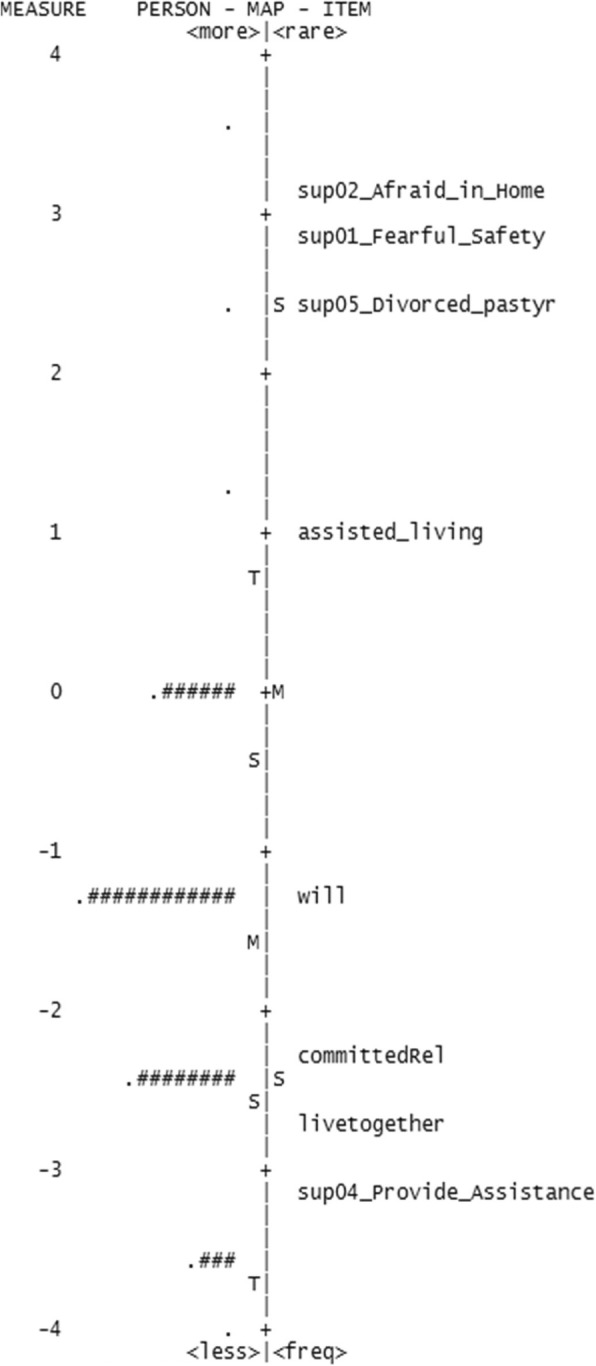
Variable map for social support

**Fig. 4:**
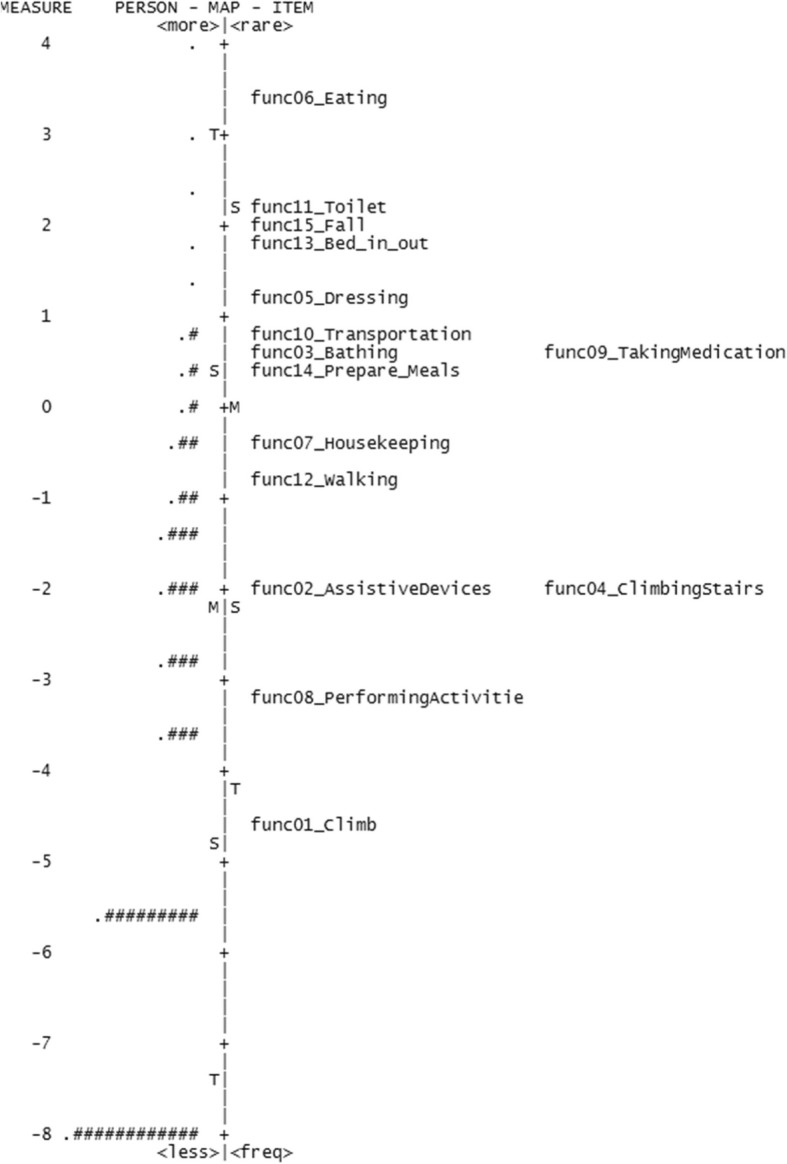
Variable map for function

However, the separation reliability values for respondents (Table 4) were unacceptably low for three of the four scales; psychological distress (0.00), symptom burden (0.50), and social support (0.00), indicating that they did not separate persons along the constructs. The separation reliability for respondents was also low for the function measure (0.75) using a cutoff of 0.80 for acceptability [28]. Figures 1, 2, 3 and 4 show the variable maps for the four constructs. The item and respondent map in Fig. 2 for psychological distress indicates that most respondents endorsed few psychological symptoms. As a result, most patients were assigned the lowest scores. The separation reliability for persons, seen in Table 4, was 0 for both “psychological distress” and “housing situation,” and 0.50 for “symptom burden” excluding extreme observations, consistent with poor separation.

**Table 4:** Respondent’s measurement results for measured (non-extreme) person

	Psychological Distress	Symptom Burden	Social Support	Function
Measures				
Mean	-1.52	-2.08	-1.50	-2.26
SD	0.92	1.47	1.13	0.46
N	1768	7754	8314	6276
OUTFIT				
Mean	0.97	0.96	1.00	0.93
SD	0.67	1.01	1.87	1.73
> 2 (count)	78	421	1008	438
INFIT				
Mean	1.00	0.99	0.95	0.88
SD	0.22	0.67	0.81	0.86
> 2 (count)	0	171	863	365
Reliability of Separation^a^	0.00	0.50	0.00	0.75

^a^The numbers in parentheses are the reliabilities computed by excluding ill-fitting examinees

In addition, the Rasch models identified items that added little additional information. Three items, (a) “no symptom(s)” from the symptom burden domain, (b) “divorced or widowed in the past year?” from the social support domain, and (c) “tendency to fall easily” from the function domain, exhibited outfit values greater than 2.0, suggesting that their information may be of little value.

### Part 2. Determination of constructs using factor analyses and cluster analysis

#### Exploratory factor analysis

A parallel analysis suggested that the 56 items could be combined into 13 factors (F). Table 5 shows the result of a nonlinear EFA model with the 13 factors that had loadings equal to or greater than 0.32. The following factors were inferred from salient item loadings: F1 ethanol consumption, F2 psychological distress or depression, F3 neurological symptoms, F5 respiratory symptoms, F6 musculoskeletal pain, F7 gastrointestinal symptoms, F8 fear, F9 housing situation, F10 dependence on assistance, F11 difficulty walking, F12 difficulty with mobility, and F13 difficulty with activities of daily living. It should be noted that items measuring fear and alcohol consumption formed their own clusters rather than clustering with psychological distress, and the hypothesized construct “symptom burden” was broken into more-specific groups (e.g., F3, F5, F6, F7).

**Table 5:** Loadings from nonlinear exploratory factor analysis with 56 items and 13 factors

	F1	F2	F3	F4	F5	F6	F7	F8	F9	F10	F11	F12	F13
1. Relatives worry alcohol consumption	0.93												
2. Need to cut down on alcohol consumption	0.71												
3. Thoughts of suicide		0.88											
4. Anxious or nervous		0.79											
5. Restless and irritable		0.89											
6. Sad most of the time		0.89											
7. Difficulty in concentrating		0.64											
8. Little interest in relationships or activities		0.67											
9. Excessive daytime drowsiness			0.32										
10. Sleep difficulty		0.42											
11. Fatigue				0.93									
12. Fearful of safety								0.81					
13. Afraid in home								0.94					
14. Tendency to fall easily			0.36							0.59			
15. Coughing					0.85								
16. Shortness of breath					0.46								
17. Wheezing					1.03		0.37						
18. Had no symptom													
19. Difficulty with pain						0.47							
20. Abnormal swelling in legs or feet			0.61										
21. Back pain						0.69							
22. Difficulty moving an arm or a leg						0.35							
23. Joint pain			0.33			0.80							
24. Joint swelling			0.54			0.62							
25. Muscle pain						0.89							
26. Changes in bowel movement							0.46						
27. Loss of appetite							0.95						
28. Nausea							0.61						
29. Diarrhea							0.72						
30. Weight loss of > 10 lbs							0.69						
31. Constipation							0.42						
32. Weight gain of > 10 lbs			0.41										
33. Difficulty swallowing							0.33						
34. Leaking urine			0.45										
35. Diminished hearing													
36. Headaches							0.36						
37. Family or friends who can assist with homecare needs													
38. divorced or widowed in the past year									-0.34				
39. In committed relationships									1.00				
40. Lives alone									1.02				
41. Lives in housing facility with assistance									0.40				
42. Living wills or advance directives													
43. Can climb two flights of stairs without stopping												0.39	0.41
44. Difficulty climbing stairs by myself												0.82	0.43
45. No difficulty performing activities of daily life												0.71	0.48
46. Difficulty walking by myself											-0.54	0.69	0.55
47. Depend on assistive devices or assistance from others in daily life										0.71	0.32		0.47
48. Difficulty bathing													0.94
49. Difficulty dressing													0.91
50. Difficulty eating													0.88
51. Difficulty housekeeping													0.80
52. Difficulty taking medications													0.86
53. Difficulty of transportation													0.85
54. Difficulty using toilet													0.90
55. Difficulty getting in and out of bed													0.90
56. Difficulty preparing meals													0.93

*Note*. Loadings equal to or greater than |0.32| are presented

Some items and factors were, on the basis of exploratory analysis, excluded from subsequent confirmatory factor and MIRT analyses. For example, “fatigue”, “diminished hearing”, “headaches”, “had no symptoms”, “family or friends who can assist with homecare needs,” “divorced or widowed in the past year”, and “living wills or advanced directives” did not form coherent clusters with other items in EFA and were excluded from further analyses. Some of the standardized loadings in Table 5 were greater than one (e.g., item 17 on F5, item 40 on F9), which could result from too many common factors extracted. Therefore, in the subsequent analyses three factors without clear structures were eliminated. Two had only two items: F10 (“afraid in home” and “depend on assistive devices or assistance from others in daily life”) and F11 (“difficulty walking by myself” which was more highly loaded on the mobility factor and “dependent on assistive devices or assistance from others in daily life”). The third, F4 consisted of only one item “fatigue”. In summary, 3 of the 13 factors were excluded.

#### Cluster analyses

**Fig. 5:**
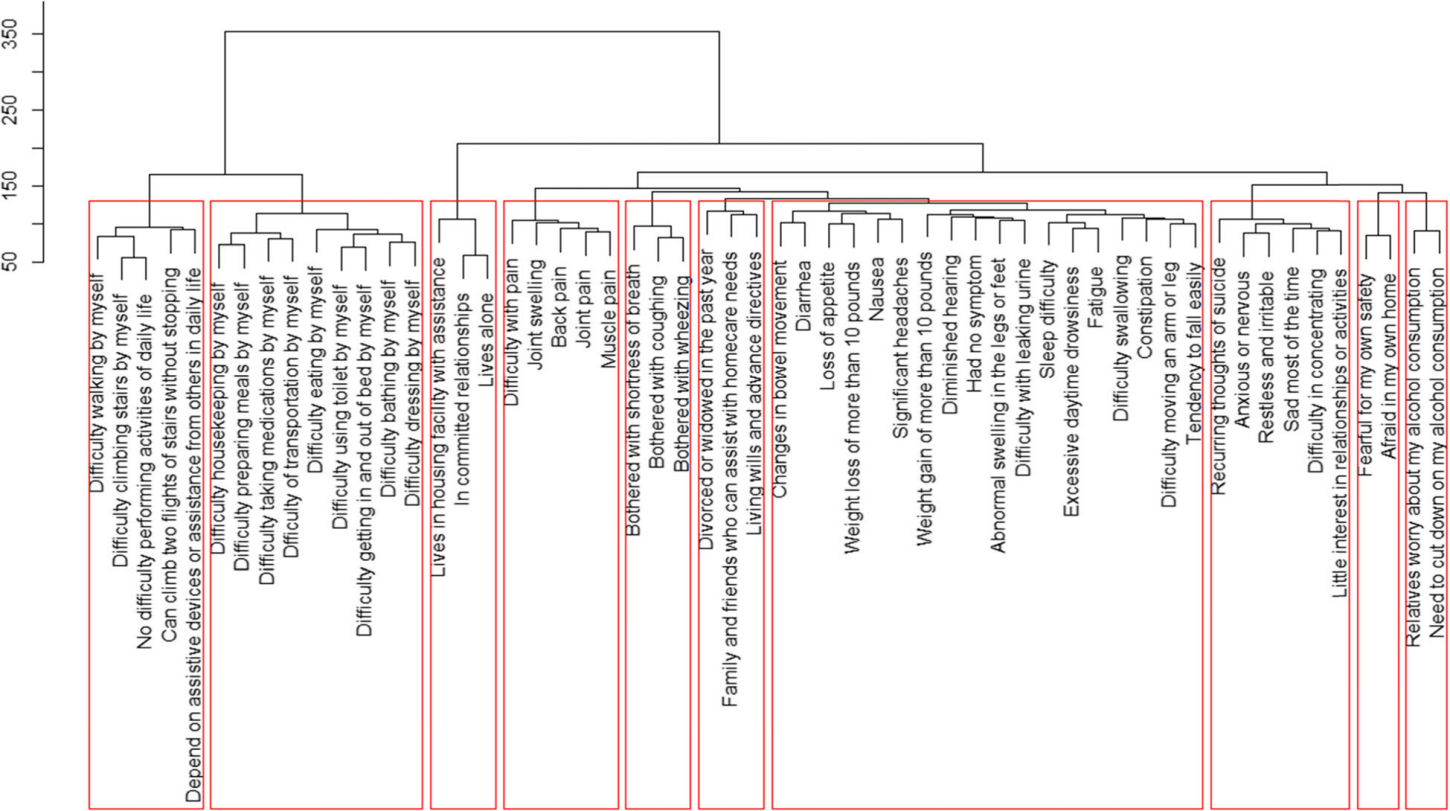
Hierarchical tree diagram from hierarchical agglomerative clustering

The findings from the hierarchical agglomerative clustering are presented in Fig. 5 and are similar to the EFA results. The following clusters, (displayed from left to right in Fig. 1) were identified: (1) mobility, (2) activities of daily living, (3) housing situation, (4) musculoskeletal pain, (5) respiratory symptoms, (6) family or social support, (7) other symptoms, (8) psychological distress, (9) fear, and (10) ethanol consumption. Unlike the case in EFA, gastrointestinal and neurological symptoms did not appear in a hierarchical agglomerative cluster solution. Conversely family or social support while it appeared in the agglomerative clustering did not appear in the EFA solution. Both methods agreed on the following domains: (1) mobility, (2) activities of daily living, (3) housing situation, (4) musculoskeletal pain, (5) respiratory symptoms, and (6) psychological distress.

### Confirmation and refinement of constructs

Reliability estimates (i.e., *α* coefficients) of the neurological and gastrointestinal symptoms were small, .59 and .54 respectively. Reliability was only .55 for the ethanol consumption and fear domains. As reliability is prerequisite to test validity [29], and also because these constructs were not identified uniformly between EFA and cluster analyses, these four constructs were removed from further analyses. The reliability of psychological distress was .70, respiratory symptom .64, musculoskeletal pain .68, housing situation .71, mobility .85, and activities of daily living .89. These latter constructs were reviewed by the expert panel for their content validity and retained for further analyses.

### Confirmatory nonlinear factor analyses were conducted using MIRT

#### MIRT analysis

**Fig. 6:**
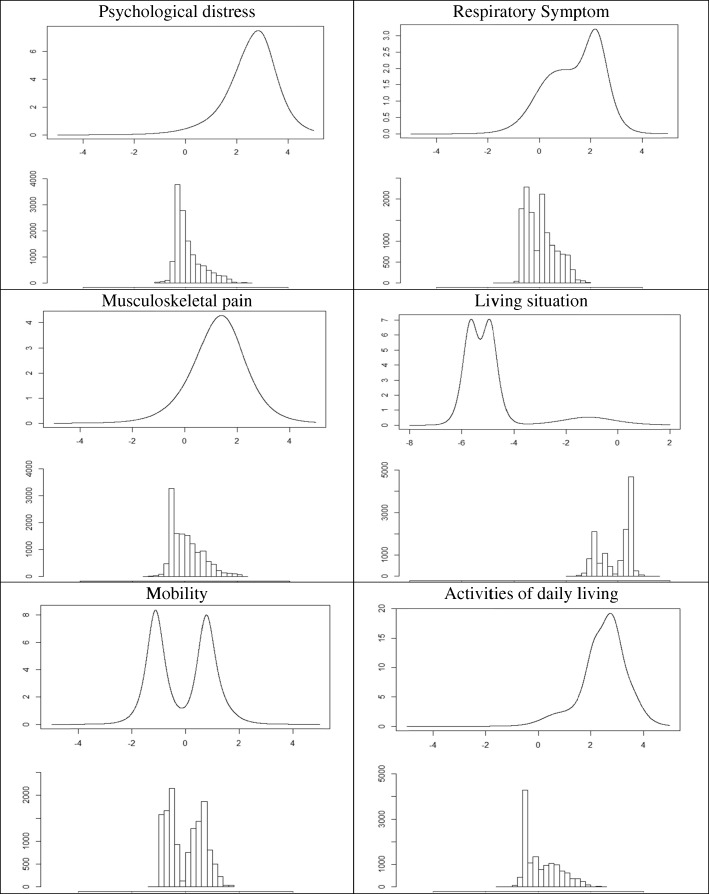
Test information curve (Up) and score distribution (Bottom) from MIRT analyses

The MIRT model included 33 items loading on six factors (i.e., Psychological Distress, Respiratory Symptoms, Musculoskeletal Pain, Family Connectedness, Mobility, Activities of Daily Living) and was fitted using the second split-half sample (*n* = 4282). This model had an excellent fit according to three fit indices (RMSR = 0.005, Tanaka fit index = 0.997, RMSEA = 0.072) although *χ*^*2*^ test of good fit was rejected (*χ*^*2*^ = 11,098.8, *df* = 480, *p* < .001). Table 6 presents item discrimination and difficulty parameters for this model. The items for psychological distress, musculoskeletal pain, respiratory symptoms, or activities of daily living were better at measuring greater rather than smaller degrees of the symptoms and problems with activities. Figure 6 shows that for each of these constructs, the peak of the information function from MIRT analyses is at the higher end of the score continuum, whereas the peak of the score density function is at the lower end. These results agree with the Rasch findings, in which psychological distress, symptom burden, and social support did not separate patients’ scores very well, because items were either almost uniformly endorsed by everyone or seldom endorsed by anyone.

**Table 6:** Item Parameters from the multidimensional IRT model with six factors

	*a1*	*a2*	*a3*	*a4*	*a5*	*a6*	*b*
1. Thoughts of suicide	1.27	0	0	0	0	0	3.67
2. Anxious or nervous	1.41	0	0	0	0	0	2.25
3. Restless and irritable	1.88	0	0	0	0	0	3.29
4. Sad most of the time	1.72	0	0	0	0	0	3.52
5. Difficulty in concentrating	1.57	0	0	0	0	0	2.89
6. Little interest in relationships or activities	1.61	0	0	0	0	0	3.07
7. Sleep difficulty	0.86	0	0	0	0	0	1.19
8. Difficulty with pain	0	0.91	0	0	0	0	0.51
9. Back pain	0	0.96	0	0	0	0	1.04
10. Difficulty moving an arm or a leg	0	1.28	0	0	0	0	1.60
11. Joint pain	0	1.01	0	0	0	0	0.93
12. Joint swelling	0	0.90	0	0	0	0	1.95
13. Muscle pain	0	1.15	0	0	0	0	1.57
14. Coughing	0	0	1.12	0	0	0	1.22
15. Shortness of breath	0	0	1.22	0	0	0	0.39
16. Wheezing	0	0	1.96	0	0	0	2.50
17. In committed relationships	0	0	0	3.00	0	0	-1.25
18. Lives alone	0	0	0	3.00	0	0	-1.75
19. Lives in housing facility with assistance	0	0	0	0.93	0	0	-1.29
20. Can climb two flights of stairs without stopping	0	0	0	0	2.11	0	1.00
21. Difficulty climbing stairs by myself	0	0	0	0	3.00	0	1.19
22. No difficulty performing activities of daily life	0	0	0	0	3.00	0	0.34
23. Difficulty walking by myself	0	0	0	0	1.72	0	1.37
24. Depend on assistive devices or assistance from others in daily life	0	0	0	0	0	2.04	1.18
25. Difficulty bathing	0	0	0	0	0	3.00	3.61
26. Difficulty dressing	0	0	0	0	0	2.09	2.98
27. Difficulty eating	0	0	0	0	0	1.10	2.81
28. Difficulty housekeeping	0	0	0	0	0	3.00	2.62
29. Difficulty taking medications	0	0	0	0	0	1.51	2.04
30. Difficulty of transportation	0	0	0	0	0	1.79	2.39
31. Difficulty using toilet	0	0	0	0	0	2.49	4.24
32. Difficulty getting in and out of bed	0	0	0	0	0	1.68	2.83
33. Difficulty preparing meals	0	0	0	0	0	2.64	2.92

*Note. a*’s denote item discrimination and *b* denotes item difficulty parameters. The numbers following *a*’s denote their respective factors

### Scoring

We computed scores based on six final constructs from the MIRT analyses rather than the four hypothesized constructs on which the Rasch analyses were conducted. We made this choice because the six constructs had adequate internal consistency estimates (at least .70 as noted above) and our expert panel agreed that the item’s contents were well aligned with their respective constructs. MIRT-based scores were highly correlated with the raw summed scores, with coefficients ranging from .85 to .98. Raw scores were also highly correlated with the weighted scores (.95 to .98) depending on the domain. Correlations between the MIRT and the weighted scores ranged from .87 to .97. The high correlations indicate that the three different scoring strategies produced scores that increased or decreased for the most part in parallel, and can be used interchangeably.

### Convergent and discriminant validity

The factor score correlations estimated from the MIRT model are presented in the lower diagonal matrix of Table 7. The strength of correlation is greater for factor scores compared to that of raw scores in the upper diagonal because the former have higher reliabilities. The pattern of correlations were as follows: Mobility and Activities of Daily Living were highly correlated, providing evidence of convergent validity, but only moderately or weakly correlated with other constructs, providing evidence of discriminant validity. Psychological Distress was moderately correlated with Respiratory Symptoms and Musculoskeletal Pain scores, and weakly correlated with Mobility and Activities of Daily Living. Being more connected with family was negatively associated with function as well as psychological and physical symptoms.

**Table 7:** Correlations among six domain scores

	Psychological Distress	Musculoskeletal Pain	Respiratory Symptoms	Living situation (family connectedness)	Mobility	ADL
Psychological Distress	1	.36	.26	-.07	.17	.14
Musculoskeletal Pain	.59	1	.21	-.08	.25	.16
Respiratory Symptoms	.48	.33	1	-.06	.26	.12
Living situation (family connectedness)	-.12	-.09	-.09	1	-.27	-.26
Mobility	.27	.37	.34	-.34	1	.62
ADL	.27	.26	.15	-.38	.84	1

*Note.* The lower diagonal contains the correlations estimated from multidimensional IRT, and the upper diagonal contains raw sum score correlations. All correlations were statistically significant at .001 level

### Known-groups comparison

Having a diagnosis of anxiety was associated with higher psychological distress MIRT scores. Diagnoses of arthritis or diseases of musculoskeletal/ connective tissues were associated with higher Pain, higher difficulty with Mobility, and higher difficulty with Activities of Daily Living scores. Diagnoses of dyspnea and diseases of respiratory system, and pulmonary symptoms were associated with higher Respiratory Symptoms scores (Table 8).

**Table 8:** Known-groups comparisonusing diagnosis variables external to the scales

	**Diagnosis of anxiety (N=2,564)**	**No diagnosis of anxiety (N=10,095)**	***Wilcoxon rank sum W*statistic**	***p*value**
Psychological Distress MIRT score	median= 0.22	median= -0.08	8,208,987	*<* .001
	**Diagnosis of arthritis (N=4,276)**	**No diagnosis of arthritis (N=8,383)**	***Wilcoxon rank sum W*statistic**	***p*value**
Pain MIRT score	median= 0.24	median= -0.14	13,694,869	< .001
Difficulty with Mobility MIRT score	median= 0.15	median= -0.23	13,969,526	< .001
Difficulty with Activities of Daily Living MIRT score	median= 0.15	median= -0.17	14,039,972	< .001
	**Diagnosis of diseases of musculoskeletal system and connective tissues (N=9,297)**	**No diagnosis of diseases of musculoskeletal system and connective tissues (N=3,362)**	***Wilcoxon rank sum W*statistic**	***p*value**
Pain MIRT score	median= 0.06	median= -0.32	11,555,660	< .001
Mobility MIRT score	median= 0.09	median= -0.38	10,897,243	< .001
Activities of Daily Living MIRT score	median= 0.08	-0.39	10,721,472	< .001
	**Diagnosis of dyspnea, diseases of respiratory system, pulmonary symptoms (N=11,476)**	**No diagnosis of dyspnea, diseases of respiratory system, pulmonary symptoms (N=1,183)**	***Wilcoxon rank sum W*statistic**	***p*value**
Respiratory symptoms MIRT score	median= 0.13	median= -0.46	4,547,788	< .001

## Discussion

Our study shows that conventional psychometric methods can be applied in a meaningful way to assess the validity of clinician-developed “home grown” screening items that are relevant to the needs and clinical care of patients with chronic disease. However, results from the Rasch analyses reveal the limited information provided by the binary screening items, even in aggregate, and their inability, apart from physical function, to discriminate trait levels in the target population. Our findings are both encouraging and sobering as they demonstrate that while conventional methods can identify constructs that discriminate in a clinically useful manner, such constructs may be limited in number-particularly if individual variations were captured more with binary than polytomous items [30]. Our results offer a cautionary tale (although perhaps not as cautionary for institutions that may have achieved more precision in their questionnaires as might be provided by instruments such as ordinal or numerical rating scales.) to others seeking to leverage and clinically apply the huge amounts of data collected with “psychometrically naïve” screening tools.

Systematic collection of patient reported information using wholly or partially “homegrown” questionnaires has been a common practice across many institutions and represents are potentially valuable sources of data to inform often overlooked dimensions of patient care. The steps followed in our effort to establish the psychometric soundness of these data are relatively straightforward, grounded in widely accepted methods. As such, they may guide others in their own efforts with similar datasets.

Our results offer several insights that may save other investigators time and resources. First, the literature search and clinician content expert inputs provided valuable information and required limited investment. Second, our multi-pronged, hypothesis-driven approach to identify constructs and items relevant a specific areas of interest (in our case hospital-based rehabilitation and supportive care) proved both effective and parsimonious. Third, EFA and cluster analyses proved useful means of assessing the robustness of the hypothesized constructs and identifying overlooked constructs. We also used reliability estimates to exclude some domains. Fourth, both MIRT and Rasch analyses showed that the items offered minimal discrimination or clinically actionable information in several domains. Fifth, all scoring approaches were essentially comparable.

Two straightforward steps following the identification of candidate constructs may have saved us time and effort; namely 1) Identifying problematic gaps in item coverage of construct subdomains, and 2) Examining the degree of variation in patients’ responses. For example, the former could have been achieved through focus groups or a modified Delphi process. Had we included this step, we may have abandoned further investigation of the social construct given that key subdomains were not represented. Regarding the second point, we noticed early on that few patients endorsed psychological distress. The Rasch and MIRT analyses subsequently confirmed a lack of variation in patients’ responses.

Despite our ability to isolate useful information, our results highlight the need for developing instruments and items with rigorous psychometric methods. Apart from function, all other scales not only lacked discrimination, but also had problematic gaps in subdomain coverage. This is partly because our form was created as a screening rather than a monitoring tool. Among the latent factors identified in this study, most function items were similar to those in the validated measures widely used for functional assessment in elderly and chronically ill populations [7, 31, 32]. This fact may explain the superior performance of the function scale, as well as its inclusion of a polytomous item which permitted a greater variability in responses.

In this paper, we focused on identifying and validating latent constructs, which can be thought of as the causes of responses to items. In the case of symptom burden originally identified by clinician experts, an alternative conception of a trait as a composite of distinct attributes may have been more sensible. That is, there may be no common cause of musculoskeletal pain and coughing, but together they could form the composite of symptom burden. In this formative model, there is no directional relationship from the constructs to the items. An example would be the FACT symptom indices, in which bone pain, coughing, and sleeping difficulty define a disease-related symptom burden [33]. In a future investigation, we could study whether a summation of all symptoms originally identified by our content experts can predict 30-day hospital readmission.

## Conclusion

The post hoc application of conventional psychometric methods can be used to aggregate and evaluate the validity of clinician-developed “home grown” PRO items that are relevant to the needs and clinical care of patients with chronic disease. This finding is important given the vast amounts of longitudinal data that have been collected from under-represented clinical and demographic patient subgroups using these tools. Unfortunately, questionnaires relying on a small number of binary screening items rather than more granular measures or with restricted content coverage may limit the information gained.

## Supplementary information

**Additional file 1.** Content of items and their response rates.

## Data Availability

The data described in this manuscript have not been de-identified and are therefore not publicly available.

## Abbreviation

ADL: Activities of daily living
CAD: Coronary artery disease
CHF: Congestive heart failure
COPD: Chronic obstructive pulmonary disease
CVI: Current visit information
EFA: Exploratory factor analysis
GED: general equivalency diploma
ICD: International classification of disease
MI: Myocardial infarction
PRO: Patient reported outcome
RMSEA: Root mean square error of approximation
SD: Standard deviation
