# Supplementary material for: Assessing whether ad hoc clinician-generated patient questionnaires provide psychometrically valid information
Source: Health Qual Life Outcomes. 2020 Mar 3;18:50. doi: 10.1186/s12955-020-01287-2 (PMC7055149; doi:10.1186/s12955-020-01287-2)
Supplement: Supplementary file 1 — Additional file 1. Content of items and their response rates. [file 12955_2020_1287_MOESM1_ESM.docx]

Supplement 1. **Content of items and their response rates**

| **Content of items and the name of the constructs (bolded) that the items were hypothesized to measure** | **Form(s) that include(s) the item** | **Complete response rates** |
| --- | --- | --- |
| **Psychological Distress/ Well being** |  |  |
| 1. Alcohol-Current Use- how often per week? | Forms A, D | 9% |
| 2. Alcohol-Current Use-None | Form A | 9% |
| 3. Alcohol-Current Use-Number of servings per day | Forms A, D | 9% |
| 4. Alcohol-Current Use-Number of years used | Forms A, D | 9% |
| 5. Alcohol-Previous Use-How often per week? | Forms A, D | 7% |
| 6. Alcohol-Previous Use-Number of servings per day? | Forms A, D | 7% |
| 7. Alcohol-Previous Use-Number of years used? | Forms A, D | 7% |
| 8. Do relatives/ friends worry about your alcohol consumption? | Forms A, B, C, D | 92% |
| 9. Do you currently use alcohol? | Forms C, D | 0% |
| 10. Recurring thoughts of death or suicide? | Forms B, C | 83% |
| 11. Have you been abused - Don’t know? | Forms A, D | 9% |
| 12. Have you been emotionally abused? | Form A | 9% |
| 13. Have you been physically abused? | Form A | 9% |
| 14. Have you been sexually abused? | Form A | 9% |
| 15. Have you ever felt the need to cut down on your alcohol consumption? | Forms A, B, C, D | 93% |
| 16. Have you felt anxious or nervous? | Forms B, C, D | 83% |
| 17. Have you felt restless and irritable? | Forms B, C, D | 83% |
| 18. Have you felt sad most of the time? | Forms B, C, D | 83% |
| 19. Have you had difficulty concentrating? | Forms B, C, D | 83% |
| 20. Have you had little interest or pleasure in relationships or activities? | Forms B, C, D | 83% |
| 21. Rate your stress level (1 lowest 5 highest) | Form A | 9% |
| 22. In an average week, minutes of moderately vigorous/ vigorous physical activity | Forms A, D | 9% |
| **Symptom Burden** |  |  |
| 23. Bothered with coughing? | Forms A, B, C, D | 92% |
| 24. Bothered with shortness of breath? | Forms A, B, C, D | 92% |
| 25. Bothered with wheezing? | Forms A, B, C, D | 92% |
| 26. Difficulty with pain? | Forms B, C, D | 76% |
| 27. Had abnormal swelling in the legs or feet? | Forms A, B, C, D | 73% |
| 28. Arthritis | Form A | 7% |
| 29. Back pain | Forms A, B, C, D | 92% |
| 30. Back stiffness | Form A | 9% |
| 31. Changes in bowel movement | Forms A, B, C | 92% |
| 32. Difficulty moving an arm or leg | Forms A, B, C, D | 92% |
| 33. Difficulty swallowing | Forms A, B, C, D | 92% |
| 34. Difficulty with leaking urine | Forms A, B, C, D | 92% |
| 35. Diminished hearing | Forms A, B, C, D | 92% |
| 36. Excessive daytime drowsiness | Forms B, C, D | 83% |
| 37. Fatigue | Forms B, C, D | 83% |
| 38. Joint pain | Forms A, B, C, D | 92% |
| 39. Joint stiffness | Form A | 9% |
| 40. Joint swelling | Forms A, B, C, D | 92% |
| 41. Loss of appetite | Forms B, C, D | 83% |
| 42. Muscle pain | Forms A, B, C, D | 92% |
| 43. Muscle stiffness | Form A | 9% |
| 44. Nausea | Forms A, B, C, D | 92% |
| 45. No symptom(s) | Form B | 83% |
| 46. Problems falling asleep | Forms A, B | 92% |
| 47. Staying asleep | Form A | 9% |
| 48. Significant Headaches | Forms A, B, C, D | 92% |
| 49. Significant problems with constipation | Forms A, B, C, D | 92% |
| 50. Significant problems with diarrhea | Forms A, B, C, D | 92% |
| 51. Vomiting | Form A | 9% |
| 52. Weight gain of more than 10 pounds | Forms A, B, C, D | 92% |
| 53. Weight loss of more than 10 pounds | Forms A, B, C, D | 92% |
| 54. Pain Scale | Form D | 0% |
| **Social Support** |  |  |
| 55. Currently married? | Form A | 9% |
| 56. Ever fearful for your own safety? | Forms B, C, D | 83% |
| 57. Ever feel afraid in your own home? | Forms B, C, D | 84% |
| 58. A living will or other advance directive? | Forms A, B, C, D | 84% |
| 59. A regular physician | Form A | 8% |
| 60. Family/friends who can provide assistance with homecare needs | Forms A, B, C, D | 91% |
| 61. Divorced or widowed in the past year? | Forms A, B, C, D | 89% |
| 62. Divorced or widowed? | Form A | 3% |
| 63. Current living arrangements | Forms A, B, C, D | 95% |
| 64. Current relationship status (check all that apply) | Forms A, B, C, D | 87% |
| 65. With whom do you live? | Forms A, B, C, D | 84% |
| **Function** |  |  |
| 66. Disabled? | Form A | 9% |
| 67. Can climb two flights of stairs without stopping to rest? | Forms B, C, D | 79% |
| 68. Depend on any assistive devices (wheelchair, cane) or assistance from other people to perform activities important in your daily life? | Forms B, C, D | 84% |
| 69. Difficulty bathing by yourself | Forms A, B, C, D | 88% |
| 70. Difficulty climbing stairs by yourself | Forms A, B, C, D | 88% |
| 71. Difficulty dressing by yourself | Forms A, B, C, D | 88% |
| 72. Difficulty eating by yourself | Forms A, B, C | 88% |
| 73. Difficulty housekeeping by yourself | Forms A, B, C, D | 88% |
| 74. Difficulty performing these activities by yourself—None | Forms A, B, C, D | 88% |
| 75. Difficulty taking medications by yourself | Forms A, B, C, D | 88% |
| 76. Difficulty transportation by yourself | Forms A, B, C, D | 88% |
| 77. Difficulty using the toilet by yourself | Forms A, B, C, D | 88% |
| 78. Difficulty walking by yourself | Forms A, B, C, D | 88% |
| 79. Difficulty getting in and out of bed by yourself | Forms B, C, D | 79% |
| 80. Difficulty preparing meals by yourself | Forms B, C, D | 79% |
| 81. Tendency to fall easily | Forms B, C, D | 83% |
| **Others** |  |  |
| 82. Highest grade or level of school completed | Forms A, B, C, D | 83% |
| 83. Current employment status (check all that apply) | Forms A, B, C, D | 87% |
